# Sjögren’s and non-Sjögren’s sicca share a similar symptom burden but with a distinct symptom-associated proteomic signature

**DOI:** 10.1136/rmdopen-2021-002119

**Published:** 2022-05-18

**Authors:** Valentina Pucino, Jason D Turner, Saba Nayar, Florian Kollert, Saaeha Rauz, Andrea Richards, Jon Higham, Ana Poveda-Gallego, Simon J Bowman, Francesca Barone, Benjamin A Fisher

**Affiliations:** 1 Institute of Inflammation and Ageing, College of Medical and Dental Sciences, University of Birmingham, Birmingham, UK; 2 National Institute for Health Research (NIHR) Birmingham Biomedical Research Centre and Department of Rheumatology, University Hospitals Birmingham NHS Foundation Trust, University of Birmingham, Birmingham, UK; 3 Department of Rheumatology and Immunology, Inselspital University Hospital Bern, Bern, Switzerland; 4 Academic Unit of Ophthalmology, Birmingham and Midland Eye Centre, Birmingham, UK; 5 Department of Oral Medicine, Birmingham Dental Hospital, Birmingham, UK; 6 Candel Therapeutics, Boston, Massachusetts, USA

**Keywords:** Sjogren's Syndrome, Autoimmune Diseases, Inflammation

## Abstract

**Objectives:**

Given the similarity in symptoms between primary Sjogren’s syndrome (SjS) and non-SjS sicca syndrome (sicca), we sought to characterise clinical and proteomic predictors of symptoms in both groups in order to better understand disease mechanisms and help guide development of immunomodulatory treatments. These have not, to date, unequivocally improved symptoms in SjS clinical trials.

**Methods:**

Serum proteomics was performed using O-link inflammation and cardiovascular II panels. SjS (n=53) fulfilled 2016 ACR/European Alliance of Associations for Rheumatology (EULAR) criteria whereas sicca (n=60) were anti-Ro negative, displayed objective or subjective dryness, and either had a negative salivary gland biopsy or, in the absence of a biopsy, it was considered that a biopsy result would not change classification status. Linear regression analysis was performed to identify the key predictors of symptoms. Cluster analysis was completed using protein expression values.

**Results:**

EULAR-Sjögren’s-Syndrome-Patient-Reported-Index (ESSPRI), EuroQoL-5 Dimension utility values, and anxiety and depression did not differ between SjS and sicca. Correlations between body mass index (BMI) and ESSPRI were found in sicca and to a lesser extent in SjS. Twenty proteins positively associated with symptoms in sicca but none in SjS. We identified two proteomically defined subgroups in sicca and two in SjS that differed in symptom burden. Within hierarchical clustering of the SjS and sicca pool, the highest symptom burden groups were the least distinct. Levels of adrenomedullin (ADM), soluble CD40 (CD40) and spondin 2 (SPON2) together explained 51% of symptom variability in sicca. ADM was strongly correlated with ESSPRI (spearman’s r=0.62; p<0.0001), even in a multivariate model corrected for BMI, age, objective dryness, depression and anxiety scores.

**Conclusions:**

Obesity-related metabolic factors may regulate symptoms in sicca. Further work should explore non-inflammatory drivers of high symptom burden in SjS to improve clinical trial outcomes.

**Key messages**

**What is already known about this subject?**

- Dryness, pain and fatigue are cardinal symptoms of the autoimmune disease Sjögren’s syndrome (SjS), as well as the less well defined non-Sjögren’s sicca syndrome (sicca).
- Clinical trials of immunomodulatory drugs in SjS have often failed to demonstrate improvement in symptoms.
- Obesity is linked with low-grade inflammation.

**What does this study add?**

- Correlations between body mass index and symptoms were found in sicca and to a lesser extent in SjS patients.
- Adrenomedullin strongly correlates with symptoms in sicca patients.
- Proteomic analysis reveals cluster stratification with symptom associations in SjS and sicca patients.
- SjS and sicca clusters associated with highest symptom burden are less distinct proteomically.

**How might this impact on clinical practice or further developments?**

- The identification of novel targets will help to identify therapeutic options for reducing symptom burden.
- The poorly defined distinction between high-symptom burden SjS and sicca subgroups support the need for better outcome measures for clinical trials.

## Introduction

Dryness, pain and fatigue are cardinal symptoms of the autoimmune disease Sjögren’s syndrome (SjS), and form the components of a validated patient-reported outcome, the European Alliance of Associations for Rheumatology (EULAR) Sjögren’s syndrome patient reported index (ESSPRI).^1^ These symptoms have a profound impact on health-related quality of life (HRQoL) in SjS.^2^ Unfortunately, clinical trials of immunomodulatory therapies in SjS that have used these symptoms as a primary outcome have often failed to show benefit over placebo. Despite the belief that B cells play an important role in SjS pathogenesis,^3^ these negative findings include phase 3 trials of rituximab.^4 5^ Similarly, a recent large phase 2b study of a novel B cell depleting agent failed to show a reduction in ESSPRI compared with placebo, despite showing a dose response in the primary outcome of systemic disease activity.^6^ One possible explanation for these discrepancies may be that the initiation but not the persistence of some SjS associated symptoms, particularly fatigue and pain, is immunologically driven.^7^ This would be compatible with observations in persistent interferon-induced fatigue and chronic fatigue syndrome.^8–10^ In addition, chronic fatigue syndrome has been associated with a metabolic signature.^11 12^

Notably, dryness, pain and fatigue are also common symptoms in non-SjS sicca syndrome (sicca), which occurs in the absence of radiotherapy and drug-induced causes and is often assumed to be non-autoimmune with heterogeneous aetiology. Such patients have been variously defined as Dry Eye and Mouth Syndrome (DEMS)^13^ or Sicca, Asthenia and Polymyalgia Syndrome (SAPS),^14^ and data suggest their functional impairment and reduction in HRQoL is the same or even greater than that seen with SjS.^15–18^ Despite this significant symptom burden and HRQoL impairment, the pathogenesis and heterogeneity of sicca syndrome is poorly characterised and treated. Given the overlap of symptoms between SjS and sicca, it is possible that there may be shared pathological processes driving symptom burden, alongside causes that are specific to each syndrome.

We, therefore, set out to take a clinical and proteomic approach to address the biological associations of these key symptoms in SjS and sicca syndrome, both to elucidate approaches to clinical outcomes in SjS but also to advance understanding and stratification of sicca syndrome.

## Methods

### Patients

The Optimising Assessment in Sjögren’s Syndrome (OASIS) cohort enrols new patients attending the multidisciplinary Sjögren’s clinic at the Queen Elizabeth Hospital Birmingham.^19^ Participants in this study were recruited between 2014 and 2019. Participants with SjS had a physician diagnosis of SjS and fulfilled 2016 American College of Rheumatology (ACR)/EULAR classification criteria for primary SjS.^20^ Sicca patients were anti-Ro antibody negative, displayed subjective and/or objective oral and/or ocular dryness, and did not have a physician diagnosis of SjS. A patient would not be classified as sicca if they had had a salivary gland biopsy result consistent with SjS (ie, focal lymphocytic sialadenitis with a focus score >1). If a patient declined to have a salivary gland biopsy, they were not included in this study if it was considered possible that a biopsy result could influence classification status. Data collected includes ESSPRI, Schirmer’s test, unstimulated whole saliva, EuroQoL-5 dimension (EQ-5D), immunological parameters (focus score, immunoglobulins, complement C3 and C4, C reactive protein (CRP), anti-Ro antibodies and rheumatoid factor (RF)), body mass index (BMI) and haemoglobin A1c (HbA1c) (table 1). EULAR Sjögren’s Syndrome Disease Activity Index (SjS group only), Hospital Anxiety and Depression Scale (HADS) and Ocular Surface Disease Index score were calculated (table 1). We also assessed the concomitant diagnosis of fibromyalgia and type 2 diabetes and whether the patients were under immunosuppressant treatment (table 1).

**Table 1:** Demographic characteristics of SJS and sicca patients

Variable	Whole cohort		Proteomic cohort	
	SjS (n=172)	Sicca (n=145)	SjS (n=53)	Sicca (n=60)
Age (years)	58.5±15	56.1±12.8	55±13	60±12
Sex	162 F 10 M	126 F 19 M	50 F 3 M	54 F 6 M
Anti-Ro positivity n (%)	149 (87)	0	41 (77)	0
Symptom duration ((years, median, (IQR))	8(8.7)	8.2 (7.5)	6 (6)	9 (7)
Schirmer’s test (mm)	6.7±9	12.5±12	9±10	15±13
Unstimulated salivary flow (mL/5 min)	0.53±0.8	0.9±1.2	0.5±0.6	0.8±1.2
Stimulated salivary flow (mL/5 min)	2.7±3.1	3.8±4.5	2.7±3	3±3
ESSDAI (1–7)	4.8±5.5	N/A	4.5±4	N/A
ESSDAI domains n (%)				
Constitutional	31 (18)		12 (23)	
Lymphadenopathy	10 (6)		2 (4)	
Glandular	46 (27)		10 (19)	
Articular	54 (31.4)		18 (34)	
Muscular	1 (0.6)		1 (2)	
Cutaneous	7 (4)		2 (4)	
Respiratory	15 (8.7)		5 (9.4)	
Neurological	9 (5.2)		2 (4)	
Haematological	37 (21.5)		9 (17)	
Biological	91 (53)		25 (47)	
Renal	7 (4)		1 (2)	
ESSPRI (0–10)	6±2	6.2±2.2	6±2.2	5.9±1.8
Focus score (<1)	1.7±0.8	N/A	1.6±1	N/A
IgG (g/L) (6-16)	17.5±9	11.2±3	16.5±7.8	10±2
CRP (mg/L) (0–3)	2.5±4.8	3.1±5.3	3.3±6.3	2±4
RF positivity n (%)	106 (62)	11 (7.6)	33 (62)	8 (13)
C3 (g/L) (0.75–1.65) C4 (g/L) (0.14–0.54)	1.3±0.3 0.3±0.3	1.4±0.2 0.2±0.1	1.4±0.2 0.2±0.1	1.3±0.2 0.3±0.1
Serum free light chain (K/L) quotient (0.26–1.65)	1.1±0.8	0.9±0.5	1.3±0.5	0.9±0.4
Hb1Ac (mmol/mol) (42-47)	39.2±8	39.2±9.7	39±10	37±6
BMI (kg/m^2^) (18.5–24.9)	27.5±5.6	28.7±6.4	28.6±6.5	28±5.8
Fibromyalgia diagnosis n (%)	15 (8.7)	29 (20)	5 (9.4)	9 (15)
Type 2 diabetes diagnosis n (%)	10 (5.8)	14 (9.6)	5 (9.4)	5 (8.3)
OSDI	42±26	47.2±26.5	38±23.4	45±24
Anxiety (HADS-A)	8.5±4.5	8.2±4.3	9.8±5	8.8±4.2
Depression (HADS-D)	7.4±4	6.9±4	8±4.4	6.6±4
EQ5D utility value (UK)	0.6±0.3	0.6±0.3	0.6±0.4	0.6±0.3
EQ5D VAS (patient)	61±20	58±22	59±22	60±20
Treatment n, (%)	52 (30) (46 HQ, 5 MTX, 2 AZA, 7 CCS, 1 MMF)	19 (13) (14 HQ, 3 MTX, 2 AZA, 3 CCS)	13 (25) (10 HQ, 1 MTX, 1 AZA, 2 CCS)	6 (10) (5 HQ, 2 MTX, 3 CCS)

Values are expressed as mean (SD) unless specified.

Normal ranges for some of the parameters are indicated in the brackets.

AZA, azathioprine; BMI, body mass index; C3, Complement component 3; C4, Complement component 4; CCS, corticosteroids; CRP, C reactive protein; EQ5D, EuroQoL-5 Dimension; ESSDAI, EULAR Sjögren's syndrome disease activity index; ESSPRI, EULAR Sjögren’s Syndrome Patient-Reported Index; HADS, Hospital Anxiety and Depression Scale; HQ, hydroxychloroquine; K/L, kappa/lambda serum free chain ratio; MMF, mycophenolate mofetil; MTX, methotrexate; n, number of patients; N/A, not applicable; OSDI, Ocular Surface Disease Index; RF, rheumatoid factor; SjS, Sjogren’s syndrome; VAS, Visual Analogue Scale.

For proteomic analysis, a subset of consecutively eligible patients comprising n=53 SjS and n=60 sicca was selected.

### Proteomic analysis

Serum samples were collected at baseline and stored at −80°C before analysis with O-link proximity extension assays (PEA, inflammation and cardiovascular II panels) comprising 184 distinct proteins involved in immunological/inflammatory, cardiovascular and metabolic pathways (cell adhesion, apoptosis, cellular response and activation, cell metabolism). Development and optimisation of PEA have been described elsewhere.^21^ Data units are log2 scaled normalised protein expression.

### Statistical analysis

Multiple regression analysis and univariate models were performed to identify the key predictors of symptoms using R or SPSS. Correlation analyses were performed using Spearman’s correlation coefficient. Comparisons between two or three groups were evaluated using non-parametric Mann-Whitney U test or one way analysis of variance test using GraphPad (Prism) V.8. P values are reported unadjusted unless specified. A p<0.05 was considered statistically significant. Cluster analysis was completed using protein expression values in R V.3.6.1. Data were scaled and centred and assessed over a range of clusters (between 2-11) using k-means clustering, hierarchical clustering with either Euclidean distance or Pearson’s correlation distance and complete linkage; ward’s method; or the average clustering method, and fast-adaptive spectral clustering using Spectrum (V.1.1).^22^ Approaches were assessed using silhouette values, gap-statistics and within/between-cluster sum of squares. Cluster stability was assessed using clustering tree visualisations. A heuristic method was used to select the clustering method and number of clusters which displayed differences in symptom burden. Spearman’s correlations between protein expression and ESSPRI scores within each cluster were completed in R and plotted using the pheatmap package (V.1.0.12).^23^

## Results

### Baseline characteristics of patients

Clinical and demographic characteristics of the whole OASIS cohort (SjS n=172; sicca n=145), and the OASIS proteomic cohort (SjS n=53; sicca n=60), are summarised in table 1. Patients were predominantly female. There were no clinically significant differences in patients selected for proteomics in comparison with the whole cohort (table 1)). Patients with sicca in the proteomic cohort were slightly older than those with SjS (p=0.02). In total, 31/60 patients in the sicca group had a minor salivary gland biopsy, all of which were negative for SjS.

### Relationship of clinical variables to symptom burden

**Figure 1:**
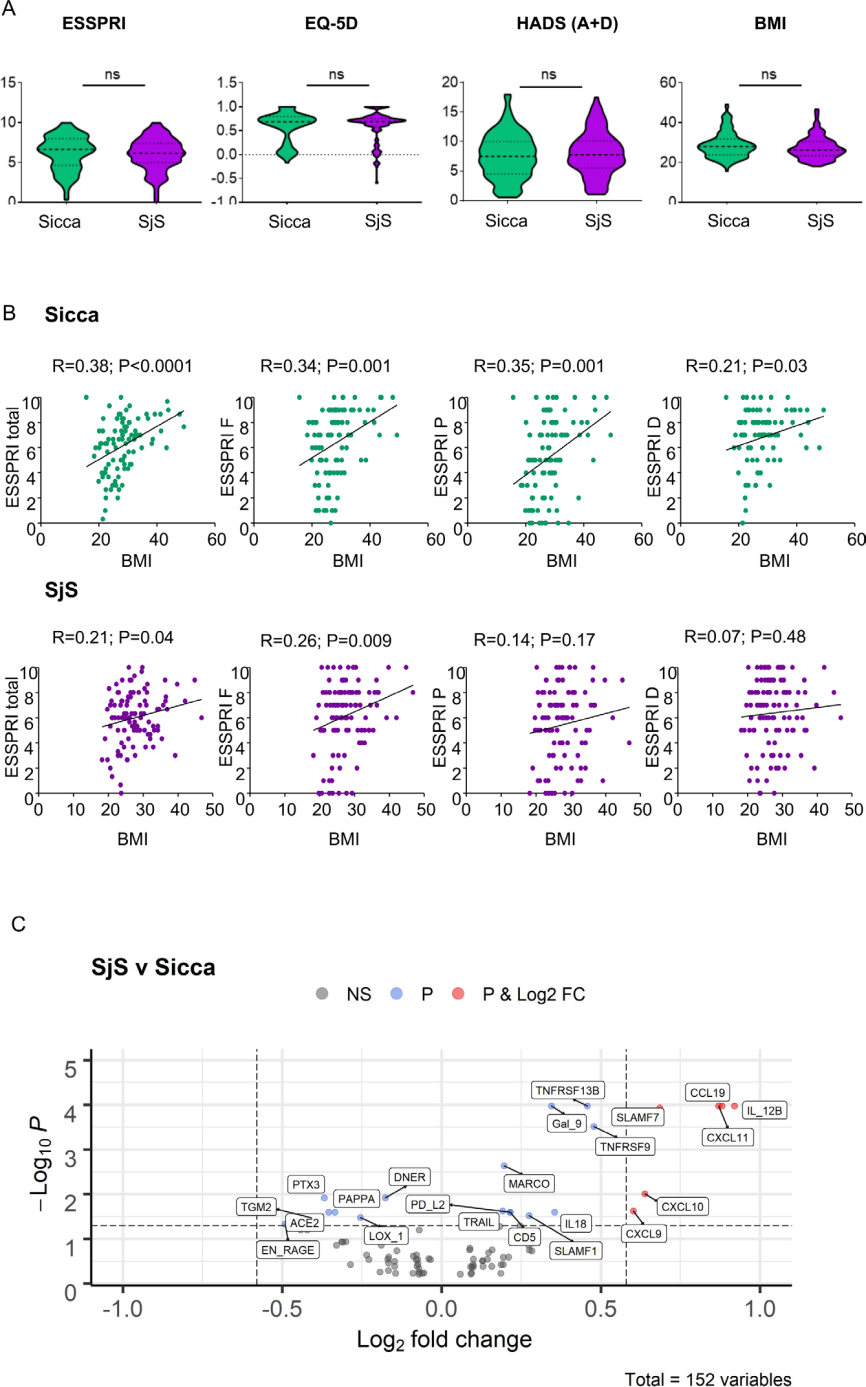
Clinical differences between SjS and sicca. (A) EULAR Sjögren’s Syndrome Patient Reported Outcome (ESSPRI), EuroQoL-5 Dimension questionnaire (EQ5D), Hospital Anxiety and Depression Scale (HADS), body mass index (BMI). Violin plots show the frequency distribution of data, median and IQR. Mann-Whitney’s U test was used for comparisons. (B) Correlation of BMI with symptoms expressed as total ESSPRI or single ESSPRI domains: fatigue (ESSPRI F), pain (ESSPRI P) or dryness (ESSPRI D) in SjS and sicca. Values represent Spearman’s correlation coefficients. P values <0.05 were considered statistically significant, ns=not significant. (C) Volcano plot showing differential regulation of proteins in SjS vs sicca. NS=proteins with a log2 fold-change between −0.58 and 0.58 and with an adjusted P value greater than 0.05. Log2 FC=proteins with a log2 fold-change greater than ±0.58 and with an adjusted P value greater than 0.05. P=proteins with a log2 fold-change between −0.58 and 0.58 and with an adjusted p value less than 0.05. P value and Log2 FC=proteins with a log2 fold-change greater than ±0.58 and with an adjusted p value less than 0.05. SjS, Sjogren’s syndrome.

Within the whole cohort there was no difference between SjS and sicca in mean ESSPRI (figure 1) or the proportion of patients with unacceptable symptom burden defined by ESSPRI≥5^24^ (n=82/172; 48%, vs n=71/145; 49%). HRQoL as measured by EQ-5D, and anxiety and depression levels assessed by HADS were also similar (figure 1A).

We analysed the predictive value of clinical and laboratory variables for ESSPRI in SjS and sicca (table 2). In both groups, ESSPRI correlated with BMI, depression, anxiety and C3, with additional associations for unstimulated salivary flow and C4 in the sicca group only (table 2). BMI and depression emerged as independent predictors of symptoms in sicca (BMI: B=0.1, 95% CI 0.03 to 0.16, p=0.005; HADS D: B=0.2, 95% CI 0.04 to 0.23, p=0.01) while BMI and anxiety were independent predictors of symptoms in SjS (BMI: B=0.1, 95% CI 0.02 to 0.16, p=0.01; HADS A: B=0.1,95% CI 0.02 to 0.24, p=0.01). BMI correlated more strongly with symptoms in sicca compared with SjS (figure 1B).

**Table 2:** Correlation of ESSPRI with clinical parameters in the whole cohort

ESSPRI versus clinical parameters	SjS (n=172)		Sicca (n=145)	
	Β (95% CI)	P value	Β (95% CI)	P value
Age	0.01 (−0.02 to 0.4)	0.37	−0.01 (−0.04 to 0.03)	0.60
HADS A	0.15 (0.07 to 0.23)	**<0.0001**	0.1 (0.02 to 0.2)	**0.02**
HADS D	0.16 (0.07 to 0.26)	**0.001**	0.24 (0.14 to 0.34)	**<0.0001**
BMI	0.1 (0.005 to 0.15)	**0.04**	0.1 (0.06 to 0.18)	**<0.0001**
Schirmer’ s test	−0.03 (−0.07 to 0.13)	0.17	0.02 (−0.01 to 0.05)	0.24
Unstimulated salivary flow	−0.2 (-0.73 to 0.37)	0.51	−0.5 (-0.92 to 0.12)	**0.01**
C3	1.8 (0.22 to 3.6)	**0.03**	2.5 (0.89 to 4)	**0.002**
C4	0.6 (−0.16 to 0.24)	0.64	5.2 (0.72 to 9.78)	**0.02**
IgG	−0.01 (−0.06 to 0.04)	0.96	0.02 (-0.11 to 0.15)	0.79
K/L free light chain quotient	−0.2 (−0.62 to 0.20)	0.31	0.3 (−0.55 to 1.11)	0.51
CRP	0.01 (−0.11 to 0.13)	0.85	0.07 (−0.03 to 0.16)	0.17

Values shown are the linear regression unstandardised coefficients and 95% CIs.

P values <0.05 marked in bold.

BMI, body mass index; CRP, C reactive protein; ESSPRI, EULAR Sjögren’s Syndrome Patient-Reported Index; HADS, Hospital Anxiety and Depression Scale; SjS, Sjogren’s syndrome.

### Differential regulation of serum proteins in SjS and sicca

Prompted by the strong association between BMI and symptoms we analysed sera of SjS (n=53) and sicca (n=60) patients for proteins relevant to both inflammation and metabolism. Six samples were discarded due to poor quality and were not considered for analysis. In the remaining samples, 152/184 proteins (86.4%) were detected above the lower limit of detection in at least 75% of the samples.

A total of 23 proteins were differentially expressed between SjS and sicca; 16 proteins with higher serum levels in SjS and 7 higher in sicca (figure 1C). Only one protein (SPON2) correlated with ESSPRI in SjS (inversely) whereas 20 proteins were positively associated with ESSPRI in sicca (online supplemental file 1), of which only 1/20 was differentially upregulated in sicca (ACE2) and 6/20 (TNFRSF13, IL-12Β, Gal9, CD5 and TNFRSF9, SLAMF1) were upregulated in SjS as compared with sicca.

10.1136/rmdopen-2021-002119.supp1 Supplementary data

**Figure 2:**
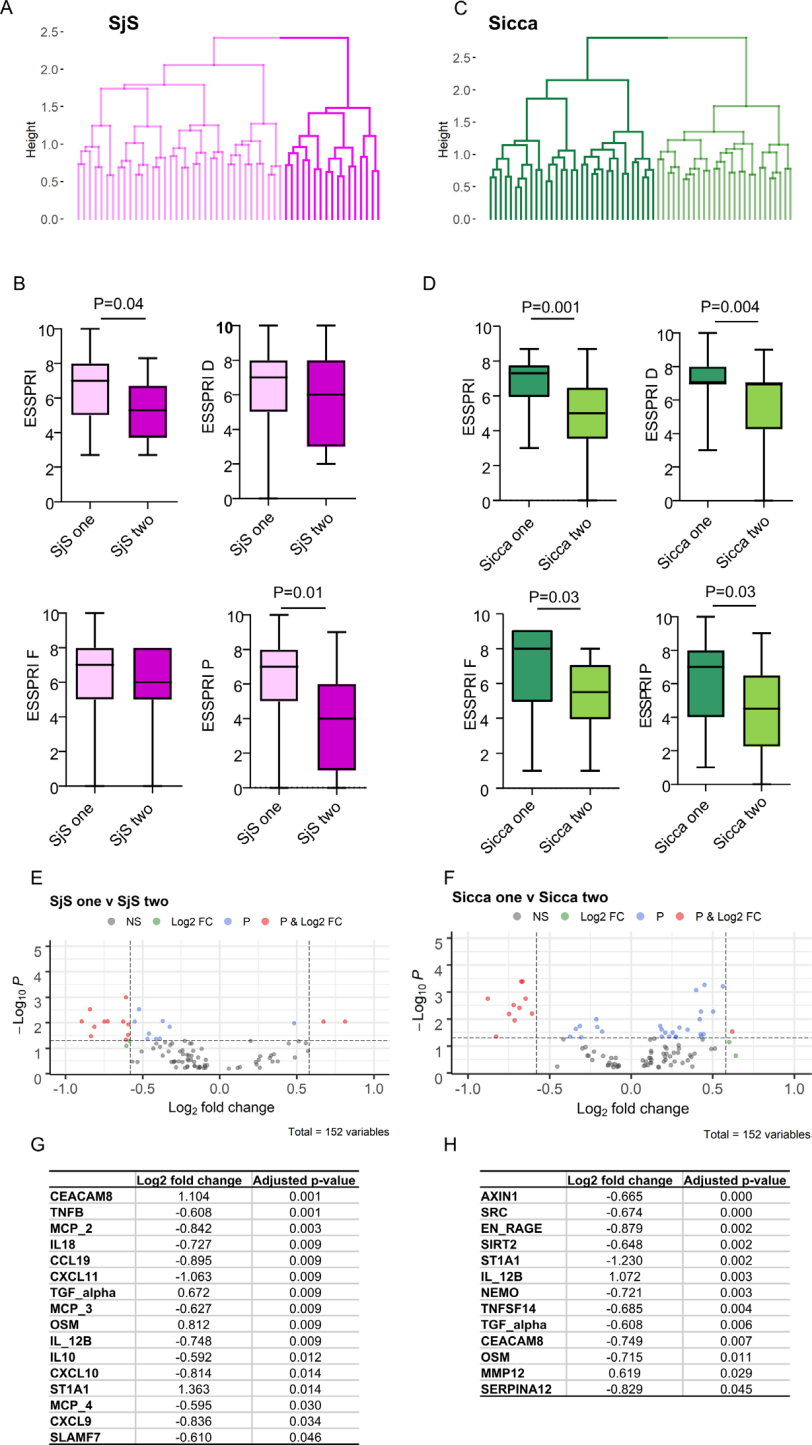
Proteomic stratification of SjS and sicca patients (A, C) Dendogram shows results of the protein derived cluster analysis of SjS (A) and sicca (C). Two clusters for SjS (SjS 1 and 2, (A) and two for sicca (sicca 1 and 2, (C) were identified. (B, D) Box plots of ESSPRI scores of the proteomic subsets of SjS (SjS 1 and 2, (B) and sicca (sicca 1 and 2, (D) identified by the cluster analysis (shown as dendrograms in A, C). (E, F) Volcano plots showing differential regulation of proteins in SjS 1 and 2 (E), and differential regulation of proteins in sicca 1 and 2 (F) subclusters. (G, H) Significant proteins are shown in the tables below the plots. NS=proteins with a log2 fold-change between −0.58 and 0.58 and with an adjusted p value greater than 0.05. Log2 FC=proteins with a log2 fold-change greater than ±0.58 and with an adjusted p value greater than 0.05. P=proteins with a log2 fold-change between −0.58 and 0.58 and with an adjusted p value less than 0.05. P value and Log2 FC=proteins with a log2 fold-change greater than ±0.58 and with an adjusted p value less than 0.05. ESSPRI, EULAR Sjögren’s Syndrome Patient-Reported Index; EULAR, European Alliance of Associations for Rheumatology; SjS, Sjogren’s syndrome.

**Figure 3:**
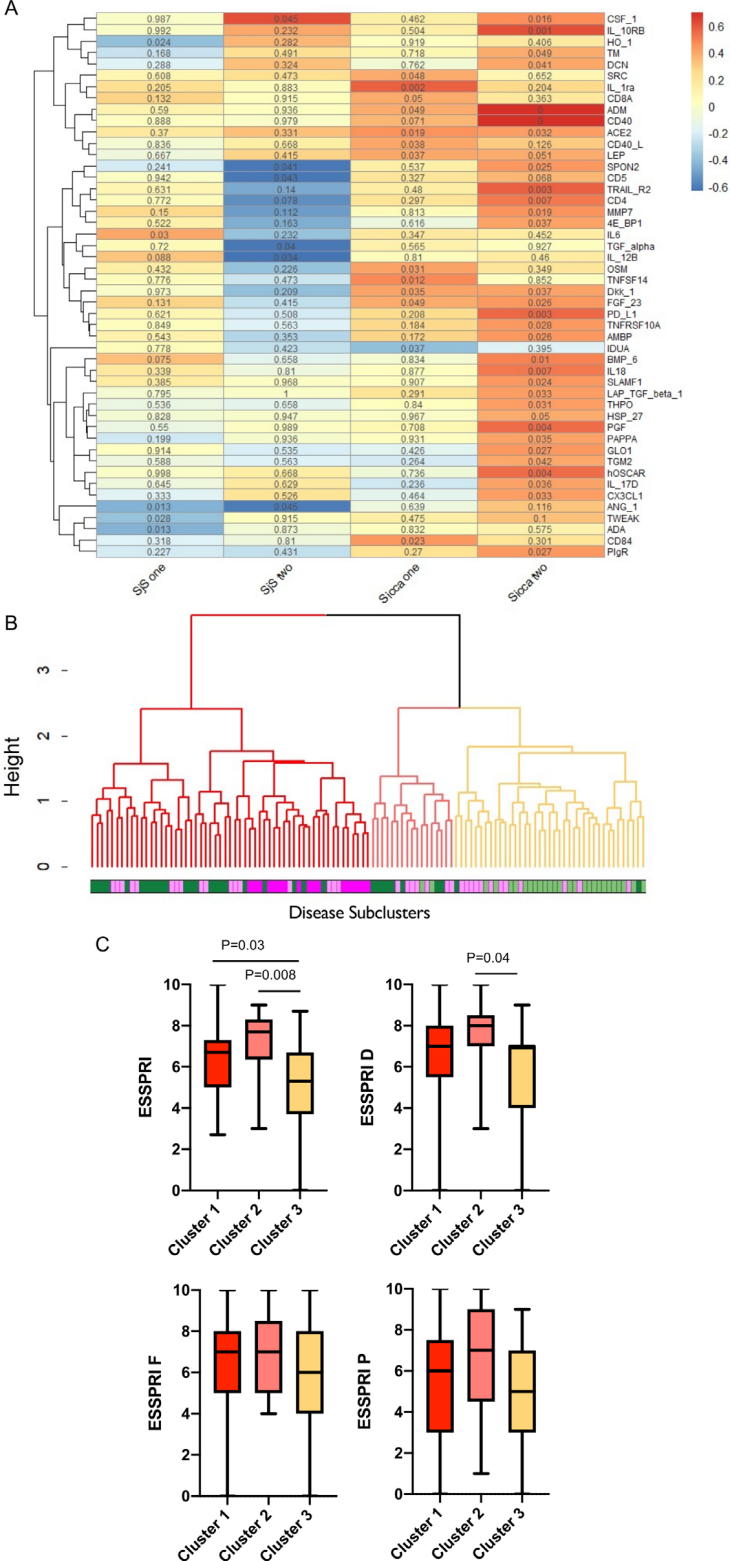
Proteomic stratification of SjS and sicca subgroups. (A) Heatmap shows protein-ESSPRI correlations within each cluster (SjS 1 and 2, sicca 1 and 2). The colour of the cells and the scale bar represent the r value, the number in each cell is the p value. (B) Dendogram showing protein derived clusters (clusters 1, 2 and 3) within the pool of SjS and sicca patients. For the disease colour bar green represents sicca and purple SjS, shades match those assigned to sub-clusters and used in figure 2A–D. The clustering indicates preservation of one SjS and one sicca cluster at the global cluster level, whereas the remaining two clusters were interspersed. (C) Box plots of ESSPRI scores of the proteomic subsets (clusters 1, 2, 3) of pooled SjS and sicca patients identified by the cluster analysis (shown as dendogram in B). Mann-Whitney’s test was used for the analysis of two groups, ANOVA test for the analysis of three groups. Values of p<0.05 were considered statistically significant. ANOVA, analysis of variance; ESSPRI, EULAR Sjögren’s Syndrome Patient-Reported Index; EULAR, European Alliance of Associations for Rheumatology; SjS, Sjogren’s syndrome.

Given the association between symptoms and SjS -upregulated proteins in the sicca group, we explored proteomic clusters within the disease groups (see the Methods section). For each disease two clusters were identified that differed in ESSPRI score (figure 2A–D), protein expression (figure 2E, F, G, H) and patterns of protein-symptom correlation (figure 3A). Further, given the similarity of symptoms between SjS and sicca, we sought to identify proteomically defined subgroups within the pool of SjS and sicca patients (figure 3B, C). One SjS and one sicca cluster showed preservation at the global cluster level suggesting disease-specific processes, whereas the remaining two clusters were interspersed (figure 3B, C). The interspersing of these SjS and sicca clusters, both of which were associated with higher symptom burden, raised the possibility of either a shared proteomic signature or else sharing of non-proteomic contributors to symptoms. However, neither of the SjS clusters had a profile of protein-symptom associations that resembled either of the sicca clusters (figure 3A).

Stepwise regression models incorporating all the symptom-associated proteins in sicca found that three proteins, adrenomedullin (ADM) (B=2.5, 95% CI 1.63 to 3.59, p<0.0001), CD40 (B=2.5, 95% CI 1.14 to 3.85, p<0.001) and SPON2 (B=−4.6, 95% CI −8.64 to 1.16, p=0.01) had an adjusted R^2^ value 0.51 implying the ability to explain 50% of variability in symptoms (online supplemental table 2).

**Figure 4:**
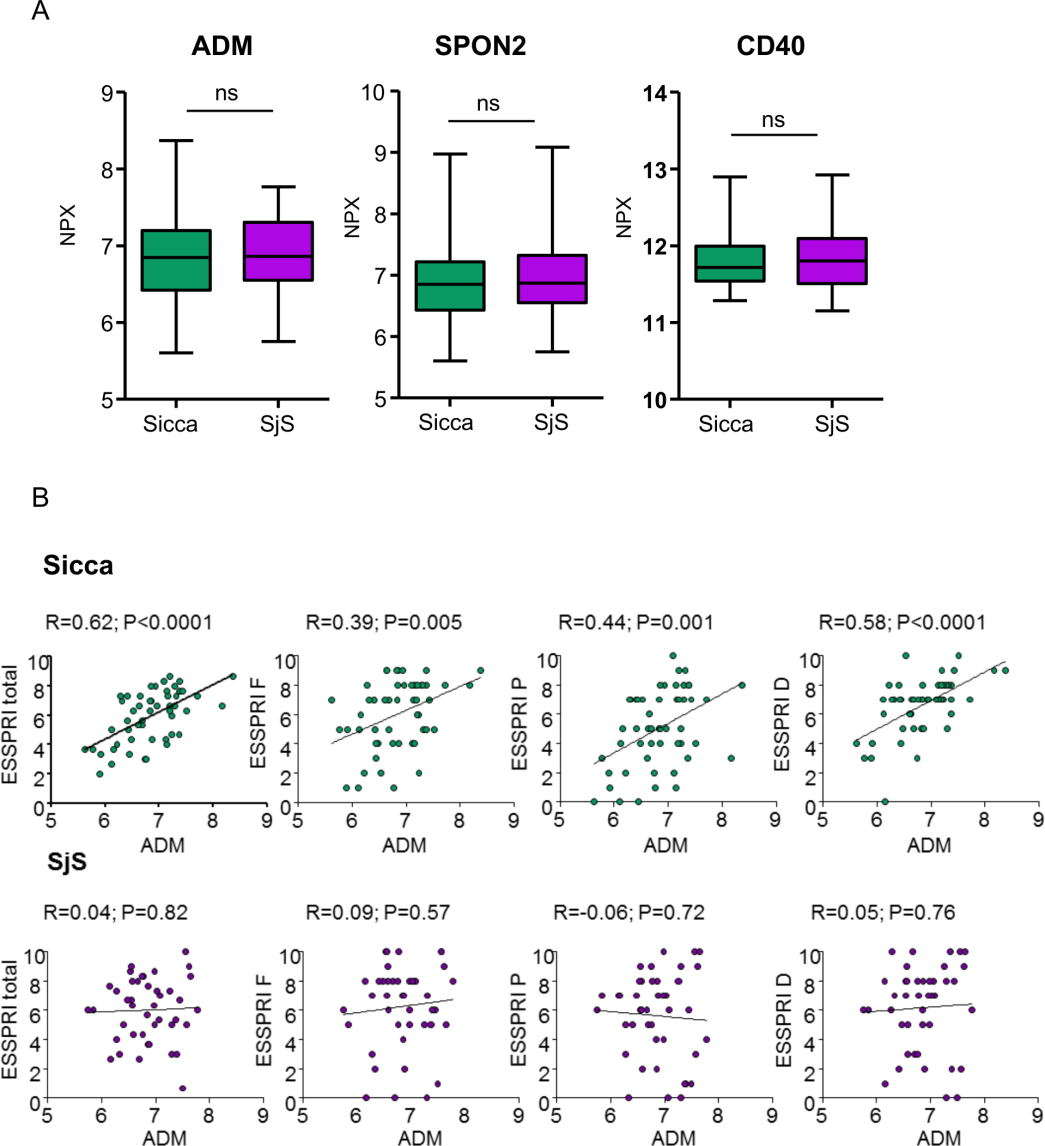
Levels of key symptom-associated proteins in sicca do not differ from SjS and ADM correlates strongly with symptoms in sicca. (A) Box plots of serum ADM, SPON2 and CD40 in SjS and sicca. NPX=normalised protein expression (log2). Mann-Whitney’s U test was used for comparisons. (B) Correlation of ADM with symptoms expressed as total ESSPRI or single ESSPRI domains: fatigue (ESSPRI F), pain (ESSPRI P) or dryness (ESSPRI D). Values represent Spearman correlation coefficients between ADM and symptoms. Values of p<0.05 were considered statistically significant. ADM, adrenomedullin; ESSPRI, EULAR Sjögren’s Syndrome Patient-Reported Index; EULAR, European Alliance of Associations for Rheumatology; ns, not significant; SjS, Sjogren’s syndrome.

Although we did not observe differences in the protein levels between SjS and sicca (figure 4A), particularly strong associations were noted between ESSPRI and ADM in the whole sicca group (but not SjS)(figure 4B) and with ADM and CD40 in the second sicca cluster.

ADM also correlated with a number of other clinical features in patients with sicca (online supplemental table 3) including BMI, which we had previously found to be associated with ESSPRI (figure 1B). However, ADM retained a strong association with ESSPRI even when correcting for BMI (ADM: B=1.9, 95% CI 1.06 to 2.80, p<0.0001; BMI:B=0.06, 95% CI −0.32 to 0.16, p=0.2). Similarly, correction for age, gender and HbA1c did not alter the association between ADM and ESSPRI (data not shown). ADM also correlated positively with triglycerides ((mg/dL), R=0.29, p=0.002), and (CRP (mg/L), R=0.24, p=0.01) and negatively with glomerular filtration rate (GFR (mL/min), R=−0.22, p=0.02) within the pool of SjS and sicca patients.

Addition of clinical variables associated with ESSPRI to the model (age, BMI, depression, unstimulated salivary flow and C3) did not further increase the ability to explain variability in symptoms (adjusted R^2^=0.53; online supplemental table 4).

Six patients in the sicca group were on immunomodulatory treatment at baseline. Removing these patients did not alter the association between ADM and symptoms (data not shown).

## Discussion

Sicca syndrome is poorly characterised with a symptom burden very similar to SjS. Clinical trials of immunomodulatory therapy in SjS have not so far had the desired impact on symptom scores and we hypothesised that analysing symptom associations in SjS and sicca together may shed light on this. We first confirmed in our cohort that patients with SjS and sicca have very similar ESSPRI scores and HRQoL impairment. This is consistent with limited data in the literature and highlights unmet need in sicca.^15–18^ Clinical predictors of symptoms, including BMI and depression/anxiety, were also similar. Next, in SjS we observed no positive associations with symptoms, as measured by ESSPRI, and a large serum protein panel that included numerous inflammatory cytokines. In contrast, we observed multiple protein associations with symptoms in sicca. Associated proteins included inflammatory mediators and metabolic/endocrine proteins, consistent with the strong association of BMI with symptoms that we observed and in line with a well-recognised relationship between obesity and inflammatory disorders.^25^

Symptom burden is a key driver of poor HRQoL in SjS and both patients and regulators expect a successful treatment to result in improvement. Yet, measurement of such symptoms might be confounded by factors not directly related to SjS, leading to measurement ‘noise’, as well as symptom-clusters in SjS that may differentially associate with pathogenic pathways.^26^ Given the similarity in symptoms between SjS and sicca, and the strong symptom associations of non-differentially expressed proteins in sicca, we anticipated that clinical and protein associations within the latter may be reflective of subgroups in SjS.

Surprisingly, although we found some overlap between SjS and sicca clusters, we did not find protein-symptom associations in either of the SjS clusters that resembled that seen in sicca. The lack of inflammatory protein associations with symptoms in SjS provides further evidence that there may be non-inflammatory counterregulatory pathways contributing to the generalised symptoms experienced by patients with SjS. The lack of a clear proteomic distinction between the SjS and sicca clusters with the highest symptom burden, also suggest that symptoms may be most uncoupled from blood measures of inflammation in SjS patients with the highest symptom burden. Clearly fatigue and pain, and to a lesser extent dryness, may have multifactorial causes. It is possible that the impact on patient reported outcomes in SjS clinical trials may be improved by rigorous exclusion of other contributors to pain such as osteoarthritis and fibromyalgia, with a focus on SjS patients with a shorter disease duration.

Sicca syndrome is poorly characterised and often considered to be heterogeneous in aetiology, but we identified surprisingly strong associations of symptoms with BMI and some proteins, especially ADM. Importantly, ADM levels were an independent positive predictor of symptom burden in multivariate models that included other symptom-associated proteins and clinical factors such as age and BMI. ADM is a vasoactive peptide belonging to the calcitonin gene related peptide family that is released during inflammation. It has additional immune regulatory and neurological functions including pain signalling.^27^ ADM has been implicated in central and peripheral pain sensitisation induced by inflammation and bone metastases.^27 28^ Intrathecal injection of an ADM receptor antagonist markedly reduced the hyperalgesia following complete Freund’s adjuvant induced inflammation in rats.^29^ In addition, ADM has been associated with renal function^30^ which can explain the inverse correlation with GFR we have observed.

Interestingly, ADM levels did not differ between SjS and sicca, however, it may be hypothesised that negative regulatory pathways that downregulate inflammation in SjS^7^ contribute to symptom burden and so efface an association between ADM and symptoms in SjS. In sicca, conversely, ESSPRI associations are seen with both ADM and selected proinflammatory cytokines. Notably, we also identified soluble CD40 as an independent symptom-associated protein in sicca. CD40 is expressed on the cell surface and functions as an immune costimulatory molecule and is a current therapeutic target for investigational drugs in SjS^31 32^; it may be shed following activation.

Limitations of our study include the relatively small number of proteins studied that excluded some inflammatory mediators of potential importance to SjS such as CXCL13^33^ and type 1 interferons, although the latter have been reported to have an inverse correlation with symptoms in SjS.^34^ Sample size is small and this may have influenced the cluster associations. We acknowledge that the ESSPRI has only been validated in a SjS population, however the items assessed are relevant to sicca syndrome and we believe the tool provides usable data in this population also.

In conclusion, sicca syndrome, or DEMS/SAPS, is a neglected syndrome with unmet medical need that requires further research. Sicca syndrome has been variously defined in past studies and work is required to establish classification to facilitate comparison across studies. Further work should examine the role of ADM in sicca, and explore non-inflammatory drivers of symptoms in SjS, to allow the identification of novel therapeutic options for reducing symptom burden and improving clinical trial outcomes.

## Data Availability

Data are available on reasonable request. All data are available on reasonable request to the corresponding author.
