# Supplementary material for: Sjögren’s and non-Sjögren’s sicca share a similar symptom burden but with a distinct symptom-associated proteomic signature
Source: RMD Open. 2022 May 18;8(1):e002119. doi: 10.1136/rmdopen-2021-002119 (PMC9121491; doi:10.1136/rmdopen-2021-002119)
Supplement: Supplementary data [file rmdopen-2021-002119supp001.pdf]

Supplementary information for the manuscript:

"Sjögren's and non-Sjögren's sicca share a similar symptom burden but with a distinct symptom-associated proteomic signature"

Valentina Pucino[1,2*], Jason D. Turner[1*], Saba Nayar[1], Florian Kollert[3], Saaeha Rauz[1,4], Andrea Richards[5], Jon Higham[5], Ana Poveda-Gallego[1,5], Simon J. Bowman[1,2], Francesca Barone[1,2,6] and Benjamin A. Fisher[1,2]

Supplementary Table 1 – Associations between proteins and symptoms (ESSPRI). Values shown are the unstandardized coefficients and 95% confidence intervals.

**Proteins vs ESSPRI**	**SjS (n = 53)**		**Sicca (n = 60)**	
	**B (95% CI)**	**P value**	**B (95% CI)**	**P value**
**ADM**	0.15 (-1.22 to 1.53)	0.83	2.2 (1.44 to 3)	**<0.0001**
**TNFRSF13B**	-0.3 (-1.5 to 0.9)	0.60	1.9 (0.64 to 3.17)	**0.004**
**FGF_23**	0.7 (-0.42 to 1.82)	0.21	1.9 (0.70 to 3.1)	**0.005**
**CD40**	-0.1 (-2.1 to 1.9)	0.92	2.9 (1.5 to 4.4)	**0.0001**
**IL_10RB**	0.35 (-1.77 to 2.48)	0.74	3.5 (1.6 to 5.4)	**0.001**
**CD5**	-0.5 (-2.65 to 1.64)	0.63	1.9 (0.53 to 3.34)	**0.008**
**CD4**	-1.3 (-3.44 to 0.82)	0.22	2.6 (1.2 to 4.04)	**0.001**
**ACE2**	0.75 (-0.48 to 1.99)	0.23	1.7 (0.88 to 2.47)	**<0.0001**
**LEP**	0.2 (-0.64 to 1.04)	0.80	0.8 (0.21 to 1.35)	**0.009**
**TRAIL_R2**	-0.04 (-1.60 to 1.52)	0.75	2.1 (0.90 to 3.33)	**0.001**

**SPON2**	-4.3<br>(-8.28 to -0.33)	**0.03**	4.3<br>(1.1 to 7.8)	**0.01**
**PD_L1**	-0.24<br>(-0.42 to 0.19)	0.42	1.35<br>(0.26 to 2.45)	**0.02**
**SLAMF1**	0.4<br>(-1.15 to 2.01)	0.73	1.4<br>(0.32 to 2.5)	**0.01**
**CSF_1**	-0.03<br>(-3 to 2.94)	0.98	4.4<br>(1.82 to 7.05)	**0.001**
**IL_1ra**	0.35<br>(-0.73 to 1.43)	0.52	0.96<br>(0.1 to 1.8)	**0.03**
**Gal_9**	-0.7<br>(-2.67 to 1.13)	0.42	2.3<br>(0.82 to 3.86)	**0.003**
**TNFRSF10A**	-0.3<br>(-1.97 to 1.35)	0.71	1.7<br>(0.37 to 3)	**0.01**
**IL_12B**	-0.02<br>(-0.98 to 0.93)	0.06	0.4<br>(-0.04 to 0.90)	**0.07**
**TNFRSF9**	0.01<br>(-1.28 to 1.3)	0.98	1.4<br>(0.39 to 2.43)	**0.008**
**TNFRSF11A**	0.01<br>(-1.51 to 1.69)	0.91	1.4<br>(0.28 to 2.91)	**0.02**

Supplementary Table 2 – Results of multiple regression analysis model (forward) for proteins and symptoms in sicca patients. Values shown are the unstandardized coefficients and 95% confidence intervals.

**Variables vs ESSPRI**	**Model (Forward)**	
	**B (95% CI)**	**P value**
**ADM**	2.5 (1.6-3.6)	**< 0.0001**
**CD40**	2.5 (1.1-3.8)	**< 0.0001**
**SPON2**	-4.6 (-8.6-1.2)	**0.009**

Supplementary Table 3 - Correlations between ADM and clinical/immunological variables. Values shown are the unstandardized coefficients and 95% confidence intervals.

**ADM versus clinical parameters**	**SjS (n = 53)**		**Sicca (n = 60)**	
	**B (95% CI)**	**P value**	**B (95% CI)**	**P value**
**Age**	0.01 (0.003 to 0.02)	**0.01**	0.02 (0.01 to 0.03)	**0.001**
**BMI**	0.03 (0.01 to 0.05)	**0.01**	004 (0.02 to 0.07)	**0.001**
**Weight**	0.01 (0.0001 to 0.01)	**0.04**	0.01 (0.003 to 0.20)	**0.01**
**Fat mass**	-0.005 (-0.003 to 0.01)	0.22	0.004 (-0.003 to 0.01)	0.25
**Free fat mass**	0.003 (-0.01 to 0.02)	0.62	0.02 (-0.003 to 0.36)	0.09
**Leptin**	0.2 (0.1 to 0.36)	**0.004**	0.3 (0.15 to 0.41)	**<0.0001**
**VAS global (patient)**	-0.003 (-0.01 to 0.005)	0.43	-0.01 (-0.02 to -0.002)	**0.008**
**EQ5D**	0.10 (-0.35 to 0.55)	0.65	-0.7 (-1.16 to -0.28)	**0.002**
**IgG**	-0.004 (-0.21 to 0.13)	0.66	0.06 (-0.01 to 0.13)	0.07
**C3**	0.7 (0.19 to 1.23)	**0.008**	0.6 (0.06 to 1.13)	**0.03**

**C4**	0.7<br>(-0.64 to 1.41)	0.45	0.6<br>(-0.25 to 2.96)	0.1
**K/L free light chain quotient**	0.01<br>(-0.12 to 0.14)	0.86	0.33<br>(0.10 to 0.71)	**0.01**
**Focus score**	0.01<br>(-0.14 to 0.17)	0.86	0.01<br>(-0.47 to 0.48)	0.98
**Schirmer's test**	-0.01<br>(-0.02 to 0.005)	0.09	-0.01<br>(-0.02 to 0.003)	0.13
**Unstimulated salivary flow**	-0.1<br>(-0.35 to 0.09)	0.24	-0.15<br>(-0.27 to -0.02)	**0.02**

Supplementary Table 4 – Multiple regression analysis models for proteins and symptoms in sicca patients. Values shown are the unstandardized coefficients and 95% confidence intervals.

Variables vs ESSPRI	Individual predictors		Model 1 (Multiple regression)		Model 2 (Forward)	
	β (95% CI)	P value	β (95% CI)	P value	β (95% CI)	P value
**Age**	0.06 (0.01-0.1)	**0.02**	-0.01 (-0.1-0.1)	0.44	-	-
**BMI**	0.16 (0.06-0.26)	**0.002**	0.06 (-0.1-0.2)	0.16	-	-
**HADS D**	0.16 (0.03-0.3)	**0.01**	0.1 (-0.1-0.3)	0.07	0.2 (0.04-0.3)	**0.008**
**C3**	1.6 (-0.9-4.2)	0.20	-0.7 (-2.9-5.6)	0.42	-	-
**Unstimulated salivary flow**	-0.6 (-1.1—0.1)	**0.02**	-0.12 (-1.7-1.8)	0.50	-	-
**ADM**	2.2 (1.4-3)	**< 0.0001**	2.3 (1.3-3.5)	**<0.0001**	2.5 (1.5-3.4)	**< 0.0001**
**CD40**	2.9 (1.5-4.4)	**< 0.0001**	2.5 (1.2-3.8)	**0.001**	2.5 (1.2-3.8)	**< 0.0001**
**SPON2**	4.3 (1.1-7.7)	0.01	-4.5 (-8--1.3)	**0.01**	-4.6 (-8.6-1.2)	**0.009**
